# Epithelial mesenchymal transition and tumor budding in aggressive colorectal cancer: Tumor budding as oncotarget

**DOI:** 10.18632/oncotarget.199

**Published:** 2010-11-28

**Authors:** Inti Zlobec, Alessandro Lugli

**Affiliations:** Institute for Pathology, University Hospital Basel, Schoenbeinstrasse 40, Basel, Switzerland

**Keywords:** colorectal cancer, tumor budding, epithelial mesenchymal transition, metastasis, oncotarget

## Abstract

Epithelial mesenchymal transition (EMT) is proposed as a critical mechanism for the acquisition of malignant phenotypes by epithelial cells. In colorectal cancer, tumor cells having undergone EMT are histologically represented by the presence of tumor buds defined as single cells or small clusters of de-differentiated tumor cells at the invasive front. Tumor budding is not a static, histological feature rather it represents a snap-shot of a dynamic process undertaken by an aggressive tumor with the potential to disseminate and metastasize. Strong, consistent evidence shows that tumor budding is a predictor of lymph node metastasis, distant metastatic disease, local recurrence, worse overall and disease-free survival time and an independent prognostic factor. Moreover, the International Union against Cancer (UICC) recognizes tumor budding as a highly relevant, additional prognostic parameter. The aim of this review is to summarize the evidence supporting the implementation of tumor budding into diagnostic pathology and patient management and additionally to illustrate its worthiness as a potential therapeutic target.

## INTRODUCTION

Epithelial mesenchymal transition (EMT) is proposed as a critical mechanism for the acquisition of malignant phenotypes by epithelial cells [1]. In colorectal cancer, tumor cells having undergone EMT are histologically represented by the presence of tumor buds defined as single cells or small clusters of de-differentiated tumor cells at the invasive front [2]. Tumor budding is predictive of lymph node metastasis, vascular and lymphatic invasion, distant metastasis, local recurrence and poor disease-specific survival time [3-15] and classified as an “additional” prognostic factor by the International Union against Cancer (UICC) [16]. Despite these highly negative attributes, surprisingly little is known about the events promoting a tumor budding phenotype although *in vitro* and xenograft animal models of EMT may provide the first clues [17-20]. The aim of this review is to summarize the evidence supporting not only the integration of tumor budding into daily diagnostic pathology and clinical management of colorectal cancer patients but also the targeting of tumor budding as a novel therapeutic approach for patients with this disease.

## EPITHELIAL MESENCHYMAL TRANSITION

EMT is a biological process allowing a polarized cell, normally interacting with a basement membrane, to assume a mesenchymal phenotype characterized by increased migratory capacity, invasiveness, increased resistance to apoptosis and increased production of extracellular matrix (ECM) components [18]. The completion of EMT is signaled by the degradation of the basement membrane and formation of a mesenchymal cell. Highly relevant for embryogenesis and wound healing, EMT has also been proposed as a critical mechanism for the acquisition of malignant phenotypes by epithelial cells [21]. EMT-derived tumor cells occurring at the invasive tumor front are thought to be those cells entering into subsequent steps of invasion and metastasis. Moreover, these cells have been shown to establish secondary colonies at distant sites that histopathologically resemble the primary tumor of origin through a process known as mesenchymal epithelial transition (MET) [21].

## HISTOPATHOLOGICAL ASPECTS OF TUMOR BUDDING

**Figure 1:**
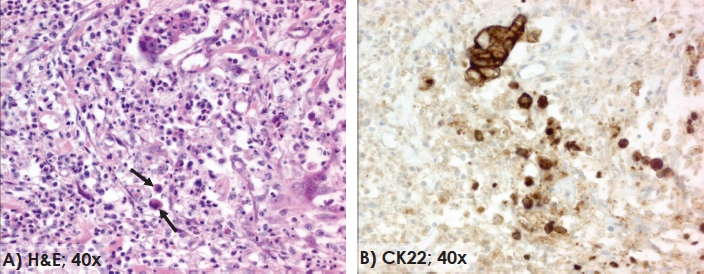
Tumor budding Single tumor buds (arrow) at the invasive front of colorectal cancer (H&E, 40x) (A). The pan-cytokeratin staining better visualizes the number of tumor buds in the same area at the invasive front (CK22, 40x) (B).

In colorectal cancer, EMT-derived tumor cells are represented histopathologically by the presence of tumor buds and are reported to occur in 20-40% of tumors [22,23]. Occurring predominantly at the invasive front, the identification of tumor buds, defined as single cells or clusters of up to 5 cells can be made using standard H&E-stained slides or facilitated by using pan-cytokeratin stains **(Figure 1)** [2]. In addition, these budding cells can often be seen in the company of “pseudo-like” cytoplasmic protrusions in direct contact with adjacent structures which are thought to be a marker of an activated budding phenotype associated with cell motility and increased invasiveness [24]. Histologically, high-grade tumor budding seems to correlate with certain parameters [25], most notably with the infiltrating tumor border configuration defined as widespread dissection of normal tissue structures with loss of a clear boundary between tumor and host tissues [23]. On the other hand, tumor budding occurs significantly less often in tumors with a more “encapsulating” or pushing/expanding growth pattern [26], itself frequently, but not always, accompanied by the presence of dense peritumoral lymphocytic (PTL) inflammation [27].

## ACTIVATION OF THE TUMOR BUDDING PHENOTYPE

The study of EMT and its related signaling pathways could provide the first clues regarding the molecular and genetics events promoting tumor budding in colorectal cancers. EMT-inducing signals from the tumor-associated stroma such as hepatocyte growth factor (HGF), epidermal growth factor (EGF), placental-derived growth factor (PDGF) and transforming growth factor-beta (TGF-beta) appear to be responsible for the induction or functional activation in cancer cells of a series of EMT-inducing transcription factors such as Snail, Slug, ZEB1, Twist, Goosecoid and FoxC2 [21,28-31]. Their implementation into the EMT program may depend on a series of intracellular signaling networks involving ERK, MAPK, PI3K, AKT, the SMADs, and integrins [32-34]. The WNT/Wingless signaling pathway, and its major effectors beta-catenin and E-cadherin are however considered integral components of EMT [21,28]. Briefly, binding of wnt proteins to a seven-span-transmembrane receptor frizzled (frz) leads to activation of WNT signaling and stabilization of cytoplasmic beta-catenin which can translocate to the cell membrane or nucleus, by mechanisms including regulation of cytokines, matrix metalloproteases (MMPs), TGF-beta, tumor necrosis factor (TNF)-alpha and HGF [35]. Membranous beta-catenin complexes with E-cadherin, a critical mediator of cell-cell adhesion and responsible for the maintenance of cell polarity [22]. In contrast, nuclear beta-catenin can function as an oncogene, binding to Tcf/LEF family members and acting as a transcriptional activator of downstream target genes [36]. Hence, membranous expression of both beta-catenin and E-cadherin characterizes the epithelial phenotype, whereas loss is indicative of a switch toward a more mesenchymal one. Up-regulation of proteins involved in ECM degradation, angiogenesis and migration such as MMP-7, MMP-26, urokinase plasminogen activator receptor (uPAR), vascular endothelial growth factor (VEGF), laminin5γ2, fibronectin and CD44 [22] have all been reported.

## IMMUNOHISTOCHEMICAL STUDES

**Figure 2:**
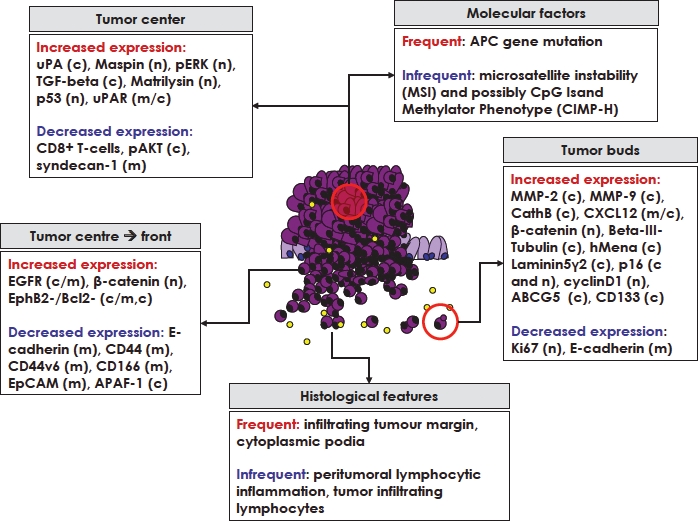
Overview of different histological features, molecular factors and protein markers linked to high-grade tumor budding Protein markers have been evaluated in the tumor centre or within tumor budding cells. Additionally, changes in protein expression from the tumor centre to the invasive front have also been related to the presence of tumor budding. Expression described predominantly as n=nuclear, m=membranous, or c=cytoplasmic. Yellow circles represent lymphocytes, in particular CD8+ T-cells.

Immunohistochemical studies have been crucial for improving our understanding of tumor budding **(Figure 2)**. High-grade tumor budding is often linked to increased expression of protein markers closely related to ECM degradation such as uPA and uPAR, matrilysin or MMPs as well as those often associated with increased proliferation such as TGF-beta, epidermal growth factor receptor (EGFR), and p53 [37-43]. Markers of cell adhesion and migration such as E-cadherin or syndecan-1 are decreased in the center of tumors with high-grade tumor budding in addition to decreased phospho-AKT, a protein reported to impact cell survival by inhibiting apoptosis [44-46]. Decreased EphB2 and Bcl-2 have been documented [47]. Interestingly, the number of CD8+ tumor infiltrating lymphocytes (TILs) is markedly decreased in high-grade budders, probably due to the relationship of the immune response with microsatellite instability (MSI) status [46]. Most interesting is the heterogeneity of expression of several markers, predominantly related to cell adhesion, from the tumor centre to the tumor front. Loss of membranous E-cadherin, CD44, CD44v6, EpCAM and CD166 expression have all been reported and often are not expressed within tumor budding cells themselves [48-51]. The finding of loss of these markers associated with more aggressive tumor behavior and high-grade tumor budding may be related to the loss of cell adhesion function which is represented by membranous staining of these markers by immunohistochemistry. Several studies have documented the changes in membranous to more cytoplasmic expression and dual-functions of proteins such as E-cadherin, EpCAM and CD44 with tumor progression, hence caution should therefore be taken to note the intra-cellular localization of these, and possibly other cell adhesion molecules [52-54].

Tumor buds themselves shows a strong and uniform nuclear beta-catenin staining and concomitant loss of membranous E-cadherin expression, in line with what is seen in EMT studies [21,55,56]. In addition, over-expression of ECM degradation proteins MMP-2 and MMP-9, uPAR, and laminin5γ2 have all been reported [41,57-59]. Additional studies have related tumor budding to increased expression of putative stem cell markers CD133 and ABCG5, as well as of beta-III tubulin, a protein involved in migration, CXCL12, a stromal cell-derived factor involved in chemotaxis and angiogenesis, hMena, a marker of cell motility and cathepsinB linked to dedifferentiation [57,60-63]. Interestingly, ABCG5-expressing and non-expressing buds have differential effects on patient survival supporting the notion that the level of aggressiveness of tumor buds may depend on their protein profiles [64]. Despite the clear association of tumor budding with migration and invasion, paradoxically, tumor buds seem to undergo low rates of proliferation as evidenced by reduced expression of proliferation marker Ki67 and concomitant increased expression of cell-cycle arrest mediators cyclin D1 and p16 [65,66].

## TUMOR BUDDING AND MICROSATELLITE INSTABILITY

Whether of sporadic or hereditary origin, tumors with high-level MSI (MSI-H; 15% of all cases), seem to have very low rates or no tumor budding [67]. In addition, *in vitro* studies comparing microsatellite stable (MSS) and MSI-H cell lines confirm the reduced EMT in the latter. Several contributing factors may help explain this finding.

### Attacker/Defender Model:

**Figure 3:**
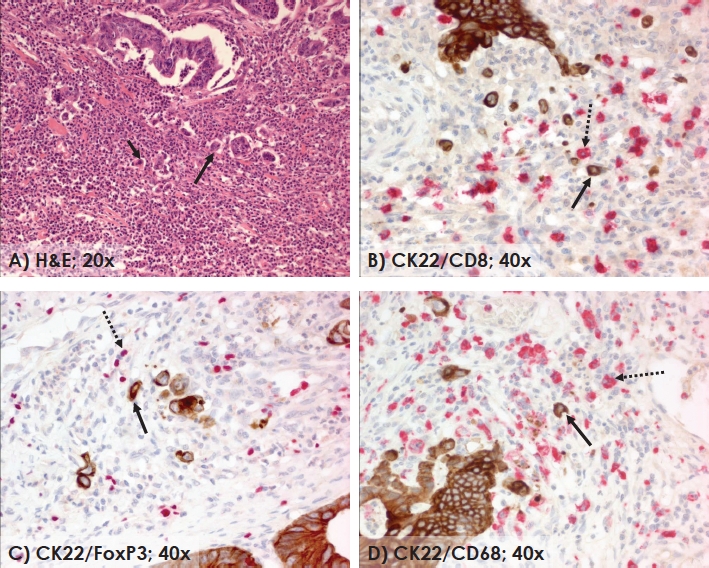
The invasive front of colorectal cancer highlighting the interaction between tumor buds and peritumoral inflammatory cells (H&E, 20x) (A). Double staining with CK22 showing presence of inflammatory cells positive for CD8 (B), FoxP3 (C) and CD68 (D) in the microenvironment of tumor buds (40x). Arrows showing examples of tumor buds (solid) and CD8+, FoxP3+ and CD68+ cells (dotted), respectively.

The invasive front of colorectal cancers can be thought of as a dynamic interface of pro- and anti-tumor factors. On the one hand, tumor buds promote progression and dissemination by attempting to penetrate vascular and lymphatic vessels. On the other, the host attempts to fend off this attack by mounting an immune response composed primarily of cytotoxic T lymphocytes, to protect vascular and lymphatic channels from invasion by tumor buds [68]. MSI-H colorectal cancers exemplify this attacker/defender model and highlight a pro-immunogenic phenotype which may to some extent be responsible for the more favorable prognosis of patients with these forms of colorectal cancers [69]. In comparison to MSS tumors, MSI-H cancers are known to have abundant CD8+ intra-epithelial and stromal TILs [70,71]. They are most often found with pushing tumor borders accompanied by dense PTL inflammation [71]. It has been previously hypothesized that specific immune responses contained within this PTL infiltrate may be targeting tumor budding cells for destruction, hence their frequent absence at the invasive front in tumors with strong lymphocytic inflammation [46]. We recently investigated the composition of the PTL infiltrate in MSI-H and MSS tumors within the tumor budding microenvironment [72]. Several differences were found including a greater number of CD8+, granzymeB+, CD16+ and CD3+ cells in MSI-H cases. Although the presence of CD8+ cells among patients with MSI-H tumors does not seem to influence outcome [73], the ratio between CD8+, FOXp3+ and CD68+ cells and the presence of tumor budding has an independent effect on prognosis in both MSS and MSI-H cancers (Figure 3). Even in cases with no obvious PTL inflammation, the higher the number of CD8+, FOXp3+ and CD68+ cells relative to the number of tumor buds (ratio of immune cells-defenders /tumor budding cells-attackers), the more favorable the impact on patient survival [72]. MSI-H cancers are known to metastasize to a much lesser degree than their MSS counterparts; the abundant immune reaction at the invasive front and particularly within the microenvironment of tumor budding cells may help further to explain this observation.

### WNT pathway signaling

**Figure 4:**
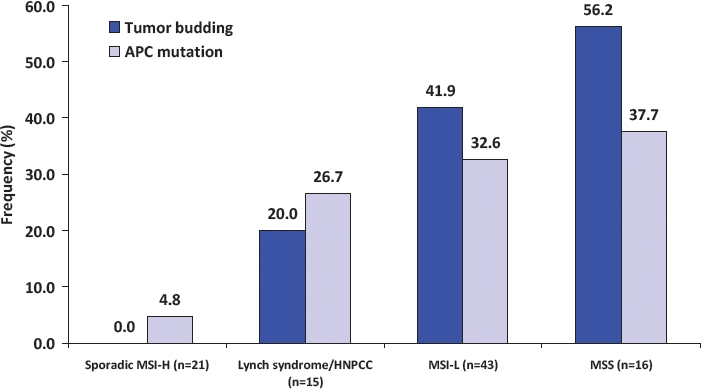
Association between APC mutation and tumor budding stratified by microsatellite instability status (adapted from Jass et al J Clin Pathol, 2003). Sporadic MSI-H colorectal cancers show the lowest rates of both APC mutation and tumor budding followed by hereditary MSI-H (Lynch syndrome; Hereditary Non-Polyposis Colorectal Cancer) cases, and low-level MSI tumors. Microsatellite stable (MSS) cancers show the greatest rates of tumor budding, accompanied by frequent APC mutation, thus substantiating the relationship between Wnt signaling and this histopathological feature.

The Wnt signaling pathway, as seen in EMT studies *in vitro*, is believed to be highly relevant to tumor budding in human colorectal cancer patients. Classically, chromosomally instable (CIN) or MSS but not MSI-H tumors may arise from inactivation of Wnt signaling [67]. Nuclear accumulation of beta-catenin is typically found in MSS colorectal cancers, occurs particularly at the invasive front and within tumor budding cells, and is simultaneously observed in cases with loss of membranous E-cadherin. MSI-H colorectal cancers typically do not show mutations in neither APC, nor present with tumor buds [67,74]. The frequency of concomitant APC mutation and tumor budding stratified by MSI status is illustrated in Figure 4 [67]. The close relationship between the two features suggests a strong interaction between inactivation of Wnt signaling and the presence of tumor budding.

### CpG Island Methylator Phenotype

MSI-H colorectal cancers have now been linked to high-level CpG Island Methylator Phenotype (CIMP-H), a feature itself strongly related to methylation of CDKN2A (p16) [75]. P16^INK4A^ is known to be a target gene of beta-catenin and often the two proteins are co-expressed within tumor budding cells [76]. Methylation of CDKN2A may lead to gene silencing and consequently decreased levels of nuclear p16^INK4a^ protein expression [77]. It is therefore expected that tumor budding should be significantly reduced in patients with CIMP-H tumors. Only a handful of studies to date have evaluated CDKN2A methylation in the context of tumor budding. Prall and colleagues found 6 cases of high-grade tumor budding with a complete absence of nuclear p16^INK4a^ protein expression (10.5%) and all had concomitant p16^INK4a^ methylation [78]. Eleven methylated cases retained expression of the protein and interestingly, cases with high-grade tumor budding often did not stain for nuclear p16^INK4a^. This lack of concordance between methylation and protein expression has been also described previously [79] and may possibly be explained by the intra-cellular localization of p16. Accumulation of cytoplasmic, rather than nuclear p16 staining has been observed within tumor budding cells [67]. Jass and colleagues hypothesized that the cytoplasmic p16 may bind cdk4 and block its translocation to the nucleus. In the absence of cdk4, cyclinD1 may complex with cdk2 thus limiting the availability of cyclins A and E and inhibiting the cell cycle which could explain the low levels of proliferation exhibited by budding cells [67,80]. Although it has been speculated that this change in intra-cellular localization within tumor buds may be due to promoter methylation of CDKN2A, the role of p16 in tumor budding in both MSS and MSI-H colorectal cancers needs further clarification. In addition, it remains interesting that CIMP-H colorectal cancers, namely those with the lowest predicted amounts of tumor budding seem to be most responsive to chemotherapy [81].

## CLINICAL USE OF TUMOR BUDDNIG

### Prognostic and predictive impact of tumor budding

Tumor budding at the invasive front has been recognized as an adverse parameter and an “additional” prognostic factor by the International Union against Cancer (UICC) [16,82]. High-grade tumor budding, irrespective of the definition, has been consistently linked to lymph node metastasis [3-8], distant metastasis [9], local recurrence [10-15] and correlates with the distance of tumor invasion beyond the outer border of the muscularis propria [83]. Tumor budding is proposed as a useful indicator of isolated tumor cells in lymph nodes in patients with node-negative colorectal cancers [84] and could indicate additional laparotomy in patients with locally excised T1 tumors [85,86].

The prognostic and independent effect of tumor budding on outcome has been investigated by several study groups. High-grade tumor budding has an independent adverse effect on both overall and disease-free survival time [23,84,87-92] particularly in the presence of cytoplasmic podia [59] and may serve as an additional histopathological parameter to identify stage I or II patients at risk of disease recurrence after curative surgery [93-96]. Even among patients with node-positive or stage III disease, tumor budding has been shown to improve the risk stratification of patients [4,97]. However, contradictory findings have been recently reported by Sy and colleagues who found an association of tumor budding with worse outcome in univariate but not multivariate analysis in this subset of patients [98]. Tumor budding may also be a predictive factor in metastatic colorectal cancer patients treated with anti-EGFR therapies [99]. In a retrospective cohort of treated metastatic colorectal cancer patients, high-grade tumor budding could predict non-response to therapy and in combination with KRAS mutational status, predicted response in 80% of cases. The predictive value of tumor budding to targeted therapy requires further investigation.

In 1989, Morodomi and colleagues published what appears to be the only work evaluating the presence of tumor budding within the tumor centre from pre-operative biopsy specimens [100]. Not only did this type of tumor budding correlate highly with budding at the invasive front, but a clear association between increased numbers of tumor buds in the pre-operative biopsy specimen and lymphatic and lymph node positivity was observed. Further studies are warranted to investigate the potential of this “intra-tumoral” type of budding as a prognostic or predictive factor in the pre-treatment clinical management of colorectal cancer patients.

### Scoring systems

Despite the clear associations of tumor budding with worse clinical outcome and more aggressive tumor parameters, tumor budding has yet to be implemented into daily diagnostic routine. The main reason for this is the absence of standardized scoring systems and sufficient evidence of inter-observer reproducibility for selected evaluation methods.

Two different types of scoring systems have been proposed: subjective and more quantitative/objective. In 1993, Hase and colleagues presented a system based on the predominant pattern of tumor budding using a 2-tier method (none or minimal versus moderate or severe) [87]. Nakamura and colleagues, using a similar system, described tumor budding along the entire invasive margin using a 4-tier method (none, mild <1/3, moderate 1/3-2/3 and severe >2/3) [9,95]. More quantitative scoring systems have been reported. The group of Ueno and co-workers proposed 2 methods by counting the number of buds within the field of most dense tumor budding: (1) using a 20x objective lens (area 0.785 mm2) and a cut-off of 5 tumor buds or (2) using a 25x objective lens (area 0.385 mm^2^) with a cut-off of 10 tumor buds. Inter-observer agreement for the latter was reported at kappa=0.84 [23. 101]. Wang and colleagues presented a technique whereby 5 randomly selected areas were evaluated, each given a score based on presence (at least one bud) or absence of tumor budding in each field (area 0.949 mm^2^) and document an inter-observer agreement of kappa=0.75 [92]. The evaluation of tumor budding cells can be significantly hindered in cases of stromal inflammation or fibrosis at the invasive front. Pan-cytokeratin immunostains facilitate significantly the visualization of tumor buds and are highly recommended for their evaluation [2]. Prall and colleagues scored pan-cytokeratin-stained tumor buds in a 0.785 mm^2^ field of vision (250x). Rather than using an arbitrary cut-off score to classify a case as “budding-positive”, they used an established statistical cut-point determination method (receiver operating characteristic (ROC) curve analysis) to identify the “optimal” number of tumor buds to be used as a threshold value [96]. Classifying tumors of ≥25 buds/field as positive, they report a strong inter-observer agreement with kappa= 0.874. Also using ROC curve analysis, our group has shown that with 15 buds/high-power field, the percent concordance between observers was 88% (kappa=0.6) [99].

These results show the potential for high-level inter-observer agreement. However, consensus has not been reached yet and international collaborative efforts to standardize scoring of tumor budding are crucial before this feature can be implemented as part of routine diagnostic pathology.

## CONCLUSION

Tumor budding is not a static, histological feature; it represents a snap-shot of a dynamic process undertaken by an aggressive tumor with the potential to disseminate and metastasize. Tumor budding is worth to be therapeutically targeted; the overwhelming and consistent evidence demonstrating that tumor budding is linked to unfavorable tumor-related features, aggressive behavior and worse overall and disease-free survival time suggests that tumor budding should be considered an “essential” prognostic factor along-side pT, pN, pM, lymphatic and vascular invasion [16]. As seen in breast and prostate cancers with the BRE and Gleason scores, respectively, tumor budding has the potential to be a basis for a supplementary prognostic scoring system in colorectal cancer once its evaluation has been standardized. The molecular and genetic events triggering a tumor budding phenotype, the changes occurring within tumor budding cells, their interaction with stromal cells and the identification of more or less aggressive tumor budding profiles remain open areas of investigation. Understanding the interactions between tumor buds and the immune response may be key toward the development of future immunotherapy targeting the destruction of tumor budding cells.
